# Cure of Recurrent Ovarian Cancer: A Multicenter Retrospective Study

**DOI:** 10.3390/cancers17183069

**Published:** 2025-09-19

**Authors:** Masahiro Sumitomo, Yasushi Kotani, Kosuke Murakami, Kaoru Abiko, Kazuko Sakai, Tomoyuki Otani, Akihiko Ueda, Masayo Ukita, Atsuko Taga, Ikuko Emoto, Kentaro Sekiyama, Minami Okudate, Motonori Matsubara, Yukio Yamanishi, Kazuto Nishio, Masaki Mandai, Noriomi Matsumura

**Affiliations:** 1 Department of Obstetrics and Gynecology, Kindai University Faculty of Medicine, Osakasayama 589-8511, Japan; 2345060904@edu.med.kindai.ac.jp (M.S.);; 2 Department of Obstetrics and Gynecology, Tenri Hospital, Tenri 632-8552, Japan; 3 Department of Obstetrics and Gynecology, Graduate School of Medical Sciences, Kanazawa University, Kanazawa 920-8641, Japan; 4 Department of Genome Biology, Kindai University Faculty of Medicine, Osakasayama 589-8511, Japan; 5 Department of Pathology, Kindai University Faculty of Medicine, Osakasayama 589-8511, Japan; 6 Department of Gynecology and Obstetrics, Kyoto University Graduate School of Medicine, Kyoto 606-8507, Japan; 7 Department of Obstetrics and Gynecology, Shizuoka General Hospital, Shizuoka 420-8527, Japan; 8 Department of Obstetrics and Gynecology, Japanese Red Cross Otsu Hospital, Otsu 520-0046, Japan; 9 Department of Obstetrics and Gynecology, National Hospital Organization Kyoto Medical Center, Kyoto 612-8555, Japan; 10 Department of Obstetrics and Gynecology, Medical Research Institute Kitano Hospital, Osaka 530-8480, Japan; 11 Department of Obstetrics and Gynecology, Kindai University Nara Hospital, Ikoma 630-0293, Japan; 12 Department of Obstetrics and Gynecology, Kobe City Medical Center General Hospital, Kobe 650-0047, Japan; 13 Department of Obstetrics and Gynecology, Toyooka Hospital, Toyooka 668-8501, Japan; 14 Department of Obstetrics and Gynecology, Japanese Red Cross Wakayama Medical Center, Wakayama 640-8558, Japan; yamanishi-y.og@wakayama-med.jp

**Keywords:** *BRCA1/2* variant, cure, disease-free survival, ovarian cancer, recurrence

## Abstract

Background: The prognosis for recurrent ovarian cancer is poor, but a small percentage of patients can be cured. The aim of this study was to clarify the criteria for being cured and the characteristics of cured cases. Methods: Ovarian cancer cases at 2 university hospitals and 8 community hospitals were analyzed to identify patients who were considered cured after complete remission (CR) following recurrence. Analyses of the tumors were performed and included *BRCA1/2* mutation analysis. Results: Of the 157 cases of recurrence, 21 (13%) showed no evidence of disease (NED). NED cases had a lower rate of ascites at the initial diagnosis, longer disease-free survival, a higher rate of solitary lesions, and a higher rate of secondary debulking surgery. All CR cases except for one showed no further recurrence when DFS reached 4 years, which was considered a criterion for being cured. The case of relapse occurred after long-term treatment with bevacizumab. Furthermore, 19.4% of the CR cases achieved 4-year DFS, which represents 9.3% of the cases of recurrent ovarian cancer and 2.3% of all cases of ovarian cancer. *BRCA* mutation analysis of the tumor was possible in 17 of the 30 cases of recurrent ovarian cancer that achieved a 4-year DFS. Pathogenic variants of *BRCA* were found in 5 of the 11 cases of high-grade serous carcinoma. Conclusions: Approximately 10% of patients with recurrent ovarian cancer achieved a 4-year DFS and were mostly cured. The curing of cases not involving high-grade serous carcinoma (HGSC) was unrelated to the presence of pathogenic *BRCA* variants.

## Simple Summary

Although ovarian cancer that comes back after treatment usually has a poor outlook, some patients may still be cured. This study aimed to find out what factors are linked to long-term survival without disease after recurrence. We looked at ovarian cancer patients from several hospitals to identify those who stayed healthy after their cancer returned. We found that about 10 percent of patients with recurrent ovarian cancer were disease-free for at least four years after complete remission. These patients tended to have fewer symptoms at diagnosis, smaller cancer spread, and more often had a second surgery. We also studied gene mutations in the tumors and found that being cured did not always depend on having a *BRCA* gene mutation, especially in cancer types other than high-grade serous carcinoma. These results may help doctors better understand which patients have a chance of long-term survival and guide future treatment plans.

## 1. Introduction

Ovarian cancer is the most lethal gynecological cancer, and its rates are increasing in Japan [1]. Ovarian cancer is often diagnosed in advanced stages with peritoneal dissemination and often recurs after a successful response to surgery and chemotherapy. Recurrent ovarian cancer has a very poor prognosis and is generally considered fatal. Nevertheless, the expectation among patients with ovarian cancer is to be completely cured [2], which may be a possibility even for recurrent cases.

For instance, a clinical trial evaluated the efficacy of secondary debulking surgery (SDS) in platinum-sensitive recurrent ovarian cancer. The trial demonstrated that after a prolonged period, the Kaplan–Meier curve for progression-free survival (PFS) reached a plateau, indicating no further recurrence [3,4,5]. In addition, clinical trials have examined the effectiveness of maintenance therapy with poly (ADP-ribose) polymerase (PARP) inhibitors for platinum-sensitive recurrent ovarian cancer. The results demonstrated that prolonged PFS is associated with a reduced incidence of subsequent progression [6,7].

Many studies have investigated factors associated with prolonged survival in recurrent ovarian cancer [8]. What patients with ovarian cancer hope for the most is to be completely cured. Nevertheless, healthcare providers often prioritize “living longer,” which conflicts with the values of patients, who mostly desire complete elimination of the cancer [2]. Healthcare providers consider it unrealistic for patients with recurrent ovarian cancer to set a goal of being cured. They believe that this objective cannot be shared unless issues such as the definition of being cured and late recurrence are resolved.

One definition of being cured of cancer is when a patient reaches the point at which disease-free survival (DFS) shows a plateau [9]. To the best of our knowledge, no study has been conducted on recurrent ovarian cancer to examine the timing of the plateau in DFS to define being cured, and no clinical trials have used being cured as an outcome. In this study, we retrospectively analyzed data from a multicenter long-term follow-up of recurrent ovarian cancer to establish a cutoff for DFS that can be considered as criterion for being cured. We examined the frequency of curable recurrent ovarian cancer and its associated clinicopathologic characteristics. The results of this study may provide treatment goals for patients with recurrent ovarian cancer and their treating physicians, as well as information for future clinical trials.

## 2. Materials and Methods

### 2.1. Study Population

Patients with epithelial ovarian cancer were included in this study. Recurrence was defined as the appearance of a clinically detectable tumor after complete remission (CR) at the end of treatment in cases where debulking surgery was performed at the time of the initial treatment. The university hospital group included cases from Kyoto University Hospital and Kindai University Hospital. Cases from Kyoto University Hospital started initial treatment between 1998 and 2015, while cases from Kindai University Hospital started between 2008 and July 2019. The final prognosis survey was conducted in July 2024. The study participants comprised 645 cases from both universities combined (Kindai: 302 participants, Kyoto: 343 participants).

The community hospital group comprised the following institutions: Tenri Hospital, Kitano Hospital, Shizuoka General Hospital, Kobe City Medical Center General Hospital, Japanese Red Cross Wakayama Medical Center, Japanese Red Cross Otsu Hospital, National Hospital Organization Kyoto Medical Center, and Toyooka Hospital. These institutions are part of the KAMOGAWA Study Group. There were 1546 cases that began treatment between 2004 and 2019. The final prognosis survey was conducted in January 2023. This study was conducted with approval of the Ethics Committee of Kindai University Faculty of Medicine (approval number R03–204). All potential study participants were allowed to decline participation at any time.

### 2.2. Disease-Free Survival (DFS)

DFS was defined as the period from the end of cytotoxic chemotherapy to the next relapse or the final follow-up date if CR was achieved through treatment. If the patient was not in CR at the end of cytotoxic chemotherapy but entered CR after receiving a PARP inhibitor, the time when CR was confirmed was used as the starting point. The DFS result after the first treatments was defined as DFS1, while the DFS result after the first recurrence was DFS2, DFS after the second recurrence was DFS3, and DFS after the third recurrence was DFS4.

### 2.3. Survey Items in the University Hospital Group

In addition to DFS, the following clinical parameters were assessed in the university hospital group. The investigated characteristics at diagnosis included age, International Federation of Gynecology and Obstetrics (FIGO) 2014 staging, histological type, CA125 level, and amount of ascites. Ascites volume was calculated from computed tomography (CT) images obtained before the initial treatment and divided into >200 mL and ≤200 mL in accordance with a previous report [10]. The treatment modalities for the primary tumor were examined, including whether neoadjuvant chemotherapy followed by interval debulking surgery (NAC-IDS) was performed, residual tumor status post-surgery, whether fertility-sparing surgery was performed, and the administration of cytotoxic chemotherapy. The characteristics analyzed at the first recurrence were the number and location of recurrent lesions, serum CA125 levels, and ascites volume (again categorized as >200 mL or ≤200 mL based on CT imaging). For the treatment of recurrent tumors, the performance of SDS and the administration of PARP inhibitors were assessed.

### 2.4. Survey Items for the Community Hospital Group

We examined whether patients had experienced a recurrence of epithelial ovarian cancer. If recurrence was observed, patients were assigned to one of the following categories: “no evidence of disease” (NED), “alive with disease” (AWD), “deceased from disease” (DOD), or “lost to follow-up or death from other causes” (Lost). For cases of NED after relapse, we collected the same clinical information as for the university hospital group.

### 2.5. Specimen Collection and Pathology Review

Formalin-fixed paraffin-embedded (FFPE) specimens were collected from cases that met the following criteria: recurrent NED, DFS of 4 years or more, and no maintenance therapy with PARP inhibitors or aromatase inhibitors. Specimens were collected from both the university hospitals and community hospitals for cases where FFPE specimens were available from tumors at the initial surgery. Central pathology review (CPR) was performed by a board-certified pathologist (T.O.).

### 2.6. Tumor BRCA Mutation Analysis

The tumor area was macrodissected from FFPE specimens and sliced into 4–5-µm sections, and DNA was extracted using a GeneRead DNA FFPE Kit (Qiagen, Hilden, Ger-many). *BRCA* mutation analysis was performed using the Oncomine *BRCA* Research Assay (Thermo Fisher Scientific, Waltham, MA) and Ion S5 XL System (Thermo Fisher Scientific). Sequencing data were imported into Ion Reporter Software version 5.18 (Thermo Fisher Scientific) for variant calling. Samples with a mean coverage of ≥300 were used for analysis. To avoid false positive results, SNVs were excluded if they had C > T/G > A mutations with <20% variant allele frequency or were detected with a low allele frequency of less than one-third of the tumor cellularity. Pathogenic *BRCA1/2* variants were determined by referring to ClinVar (https://www.ncbi.nlm.nih.gov/clinvar, accessed on 17 September 2025) and *BRCA* Exchange (https://brcaexchange.org/, accessed on 17 September 2025) [11], in addition to frameshift and nonsense variants.

### 2.7. Statistical Analysis

A comparison was conducted among three patient groups: (1) those with recurrence who did not achieve CR after initial treatment (non-CR), (2) cases of DOD and AWD (DOD/AWD), and (3) those with recurrence who achieved CR and still had NED status (CR-NED). Univariate analysis was performed using Fisher’s exact test, and multivariate analysis was performed using ordinal logistic analysis with EZR software 2.8-0 [12]. Kaplan–Meier curves were analyzed using Prism 10.4.1 (GraphPad Software, Boston, MA, USA). Results with *p <* 0.05 were considered statistically significant.

## 3. Results

### 3.1. University Hospital Groups

**Figure 1:**
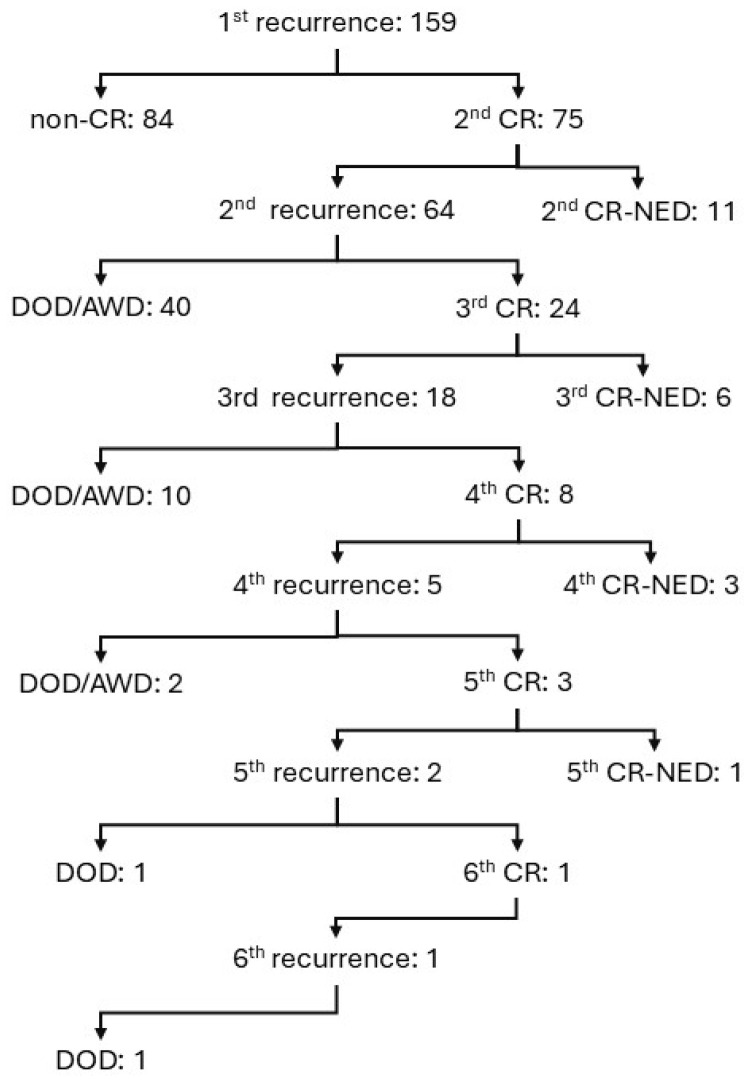
Clinical course of 159 cases that had a complete remission after initial treatment but then recurred.

Among cases from the university hospitals (Table S1), the 10-year recurrence-free survival (RFS) rate was 49.7%, and the 10-year overall survival (OS) rate was 51.9% among the 645 patients who underwent debulking surgery (Kindai: *n =* 302, Kyoto: *n =* 343) (Figure 1). Figure S1 illustrates the clinical course of patients who experienced recurrence after achieving CR following initial treatment. A total of 159 cases of recurrence were observed, and 84 of these cases (53%) did not achieve CR following treatment for recurrence (non-CR). 21 cases (13%) were considered as CR-NED, which accounted for 3.3% (21/645) of all ovarian cancer cases that underwent debulking surgery. The median follow-up duration for the 21 patients who were NED after recurrence was 12.0 years (range: 6.3–23.1 years) from the initiation of initial treatment and 9.5 years (4.2–18.7 years) from the first recurrence (Figure S2).

The patient background, initial treatment, and status at the time of the first recurrence were compared among the three groups of patients at the time of initial diagnosis (non-CR, DOD/AWD, and CR-NED; Table 1). In the univariate analysis, significant differences were observed in the proportion of patients with ascites volume > 200 mL at initial diagnosis (52%, 28%, and 12%; *p =* 0.001), the proportion of patients who did not receive chemotherapy (0%, 0%, and 14%; *p =* 0.002), the duration of DFS1 at first recurrence (median: 8.0, 13.0, and 22.8 months; *p <* 0.001), serum CA125 levels at recurrence (median: 96.0, 51.0, and 75.5 IU/L; *p =* 0.04), and the proportion of solitary recurrent lesions (13%, 29%, 52%; *p =* 0.004). In the multivariate analysis, ascites volume > 200 mL at diagnosis (*p =* 0.02), DFS1 at recurrence (*p <* 0.001), and the proportion of solitary recurrent lesions (*p =* 0.001) remained statistically significant. In addition, a subset of the cases in Table 1 limited to those with high-risk histological types (serous carcinoma and clear cell carcinoma) is listed in Table S2.

The treatments administered for recurrent tumors were compared among the groups (Table 1). Direct comparisons of treatment regimens were complicated by the non-CR group’s inclusion of cases in which adequate treatment could not be administered due to poor performance status. Therefore, the analysis was restricted to cases that had achieved CR after recurrence. Among these cases, the proportion of patients who underwent complete cytoreductive surgery during SDS was significantly higher in the CR-NED group (62%) than in the DOD/AWD group (35%) (*p =* 0.04).

When we examined the change in DFS in cases of repeated recurrence, DFS2 was shorter than DFS1 (*p <* 0.001), but there was no significant difference between DFS3 and DFS2 (Figure S3). Among cases that exhibited repeated relapses, those with a longer DFS3 than DFS2 included cases of high-grade serous carcinoma (HGSC) treated with olaparib as maintenance therapy (DFS3: 3.3 years), endometrioid carcinoma treated with bevacizumab as maintenance therapy (DFS3: 3.0 years), and low-grade serous carcinoma treated with letrozole as maintenance therapy (DFS3: 2.5 years).

**Figure 2:**
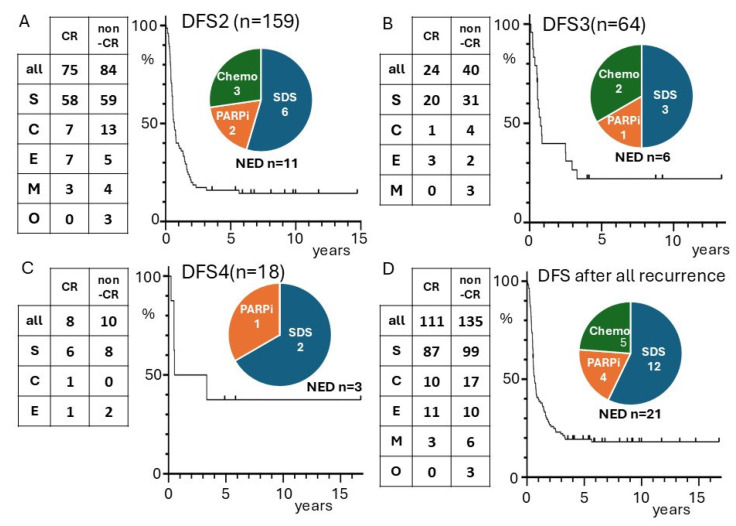
The number of cases that achieved CR after recurrence and the number of cases that did not achieve CR (non-CR), as well as the histologic type and DFS of cases that achieved CR after recurrence are shown in the Kaplan–Meier curve. For cases that had residual tumor at the start of PARP inhibitor (PARPi) treatment and achieved CR after PARPi treatment, DFS from the time of CR is shown. The pie chart shows the treatments that led to salvage in cases with NED. Cases with SDS are classified as SDS, cases with PARPi are classified as PARPi, and cases with chemotherapy only are classified as chemo. There were no cases with both SDS and PARPi. (**A**) DFS2: DFS for cases that achieved CR after the first recurrence. (**B**) DFS3: DFS of cases who achieved CR after second recurrence. One of the NED cases was classified as Chemo because measurable disease developed after SDS R0 and was salvaged by chemotherapy. (**C**) DFS4: DFS for cases that achieved CR after third recurrence. (**D**) DFS to the first recurrence event in cases that became CR after recurrence. In cases that have recurred more than twice, each DFS is counted as a separate event. S: serous, C: clear cell, E: endometrioid, M: mucinous, O: others. SDS: secondary debulking surgery.

The Kaplan–Meier curves for DFS were examined for cases that achieved CR after recurrence (Figure 2). The curve for DFS2 of cases with a second instance of CR (*n =* 75) reached a plateau at 4 years, and 11 cases were free of disease at 5 years (16%). Of these 11 cases, only one experienced a second recurrence after 5.6 years. This patient achieved CR after 9 courses of paclitaxel + carboplatin + bevacizumab following the first recurrence and then received 72 cycles of bevacizumab maintenance therapy, which was terminated due to the appearance of hypertension and proteinuria. After the bevacizumab was terminated, the patient experienced a second recurrence 1 year 4 months later. Among the 11 patients with NED after a second instance of CR, 6 patients received SDS as treatment after the first recurrence, 2 patients received olaparib, and 3 patients received chemotherapy alone (Figure 2A). Among the 2 patients who received olaparib, there were measurable tumors at the start of the olaparib treatment, but they subsequently disappeared.

The DFS3 of the 24 patients with a third instance of CR was examined, of which 6 patients were NED, including the patient who relapsed after long-term bevacizumab treatment (DFS3: 0.5 years). The rate of DFS3 of 5 years was 21% (5 patients). The Kaplan–Meier curve of 64 patients for DFS3 reached a plateau after 4 years, and no further recurrences were observed. The treatment of the 6 patients with a third case of CR in the CR-NED group included SDS in three cases, PARP inhibitors in one case, and chemotherapy alone in two cases (Figure 2B). The same investigations were also performed for the eight patients in the fourth-CR group. The Kaplan–Meier curve for DFS4 also reached a plateau after 4 years (Figure 2C).

**Figure 3:**
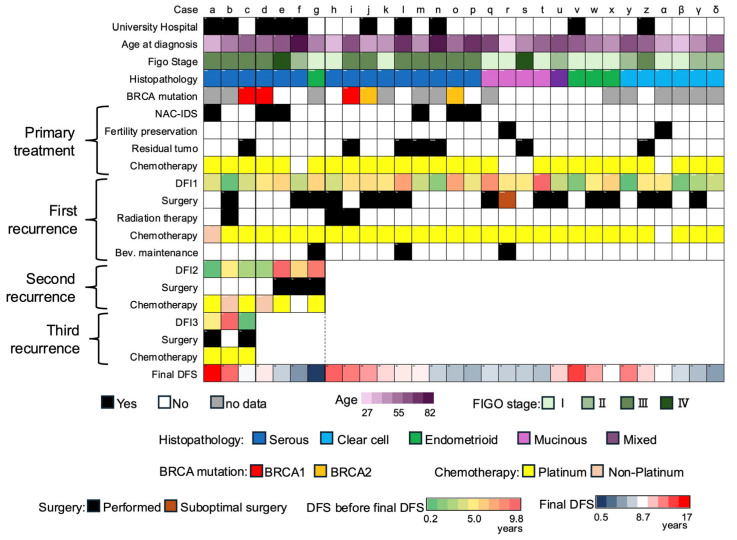
Heatmap of 30 cases with collected FFPE samples. Each column represents one case; columns are sorted by number of recurrences, histologic type, and Last DFS.

The 75 patients who achieved a second CR had a total of additional 90 recurrences and 36 CRs during their clinical course. The DFS rates for each recurrence-based event is illustrated in the Kaplan–Meier curve (Figure 2D). The DFS rate was 19.4% at 4 years, and there were no recurrences other than the case mentioned above. This rate corresponds to 9.3% of the cases of recurrent ovarian cancer and 2.3% of all ovarian cancer cases. The treatments administered at the time of the last recurrence before achieving CR-NED included SDS in 12 cases, PARP inhibitor in 4 cases, and chemotherapy alone in 5 cases. No cases were treated with both SDS and a PARP inhibitor. As shown in Figure 3, most cases treated with SDS underwent chemotherapy, but none received PARP inhibitors. Similar analyses are presented in Figure S4 for high-risk histological types (serous carcinoma, clear cell carcinoma) among cases of recurrence.

### 3.2. FFPE Specimens, CPR, and BRCA

Of the 1546 cases in the community hospital group, 53 cases (3.4%) were NED after recurrence. Of the total of 2191 cases including the university hospital, 74 cases (3.4%) were NED after recurrence. Of these, 30 cases had a DFS of 4 years or more, no history of treatment with PARP inhibitors or aromatase inhibitors, and available FFPE specimens (Figure S5). The information for each of these 30 cases is shown in a heat map in Figure 3 (cases a–d). The CPR results are shown in Figure S6.

All serous carcinomas were HGSCs, and neoadjuvant chemotherapy with interval debulking surgery (NAC-IDS) was performed for six cases of HGSCs at the time of initial treatment. Two cases (s, u) underwent fertility-sparing treatment, and peritoneal dissemination was identified as the site of recurrence in both cases. Local therapy by surgery or radiation therapy was performed at some point after recurrence for 20 cases (67%), including one case in which SDS was a suboptimal surgery (case u). Seven cases (cases a–g) exhibited more than one recurrence, and all of them involved HGSCs. One case (case g) experienced recurrence following the completion of long-term bevacizumab treatment (described above), and the last DFS for case g was 0.5 years (196 days). The last DFS for the other cases ranged from 4.1 to 16.8 years.

For the majority of cases, the operations were performed more than 10 years prior, so the DNA quality of some tumor samples did not meet the required criteria (see Methods). Thus, *BRCA* mutation analysis was feasible for only 17 cases (57%). Pathogenic *BRCA1/2* variants were identified in 5 cases, which all involved HGSCs (Table 2). Germline *BRCA1/2* variant testing was performed in only one case (case d) among the 30 cases of recurrent ovarian cancer that achieved a 4-year DFS. The same *BRCA*2 variant was detected in both the tumor and germline samples from this patient.

## 4. Discussion

Factors associated with survival beyond 5 years after recurrence of ovarian cancer include negative ascites cytology at the initial treatment [13], longer recurrence-free intervals [8,13], solitary recurrent lesions [13], and SDS [8,14,15]. In addition, long-term NED survival in recurrent ovarian cancer has been reported to be associated with a combination of local therapy and chemotherapy, including SDS [16,17] and radiotherapy [18,19]. In the present study, analysis of the three groups (non-CR, AWD/DOD, and CR-NED) demonstrated that ascites volume ≤ 200 mL at the initial treatment, longer DFS1 before the first recurrence, and solitary recurrent lesions were independent factors for NED survival (Table 1).

In addition, complete SDS after recurrence was performed more frequently in the NED group (Table 1), which is consistent with previous reports. One case of ovarian and para-aortic lymph-node recurrence after fertility-sparing treatment was included in the NED group (Table 1). Survival outcomes of recurrent cases after fertility-sparing treatment have been reported to be better than those after standard treatment [20]. DFS2 was shorter than DFS1 in cases with multiple recurrences (Figure S1), which is consistent with previous reports indicating a progressively shorter treatment-free interval with each recurrence [21]. However, DFS3 was not shorter than DFS2. This was probably due to the administration of maintenance therapy at the time of the second recurrence.

Bevacizumab is a drug that targets vascular endothelial growth factor (VEGF), and its main effect is the inhibition of angiogenesis and suppression of tumor growth [22]. Therefore, if bevacizumab is discontinued after long-term administration as maintenance therapy, the tumor cells that were present before maintenance therapy may proliferate and cause recurrence [22]. On the other hand, because PARP inhibitors have cytotoxic effects, it is possible that the tumor cells that were present before administration may be completely eliminated during long-term maintenance therapy, and the patient may be cured [23].

In “Study 19,” maintenance olaparib or a placebo was administered to platinum-sensitive cases of recurrence, and progression was reduced after 2 years in the olaparib group [6]. In the ARIEL3 study, maintenance rucaparib or a placebo was given for platinum-sensitive recurrence, and of the patients who received rucaparib and were progression-free at 44 months, 92% (36/39) remained progression-free thereafter [7]. In the present study, 4 of the 21 cases that were NED had residual lesions at the end of chemotherapy but achieved NED after subsequent olaparib administration (Figure 2A). This finding suggests that PARP inhibitors may induce remission in recurrent lesions.

With the exception of one case (Figure 3, case g), which showed recurrence after long-term use of bevacizumab was discontinued, there was no recurrence after 4 years or more of DFS (Figure 2D). This finding is consistent with data from previous clinical trials. The GOG213 study evaluated the efficacy of adding bevacizumab to paclitaxel and carboplatin (TC) and administering it as maintenance therapy for platinum-sensitive recurrent ovarian cancer. The 4.5-year PFS rate (equivalent to the 4-year DFS rate) for the TC + bevacizumab group was 9%. After one case of progression, the 5-year PFS rate was 8%, while the 4.5-year PFS rate for the TC group was 5%, and there were no further disease-progression events in either group [24].

In the GOG213 study, bevacizumab was administered to 72% of patients in the cohort, who were randomized according to the presence or absence of SDS. The 4.5-year PFS rate for the SDS group was 17%, and the 6-year PFS rate was 14%. In contrast, the 4.5-year PFS rate in the no-SDS group was 9%, and there was no further progression in either group thereafter [3].

The SOC-1 study randomized patients based on the presence or absence of SDS, and bevacizumab was not administered. The 4.5-year PFS rate was 22% in the SDS group and 7% in the non-SDS group, and there was no subsequent progression in either group. Based on the data from the present study, as well as the GOG213 and SOC-1 studies, the cutoff point for DFS at which a patient can be considered to be cured (mostly) achieved is 4 years (4.5 years for PFS) when bevacizumab is not used and approximately 2 years later if bevacizumab is used.

On the other hand, in the DESKTOPIII study, which randomized patients with or without SDS, bevacizumab was not used, but some cases recurred after 4.5 years [5]. The primary endpoint of that study was OS. The reason for these results is considered to be the absence of standardization in the timing and methods of imaging examinations to assess disease progression. However, the employment of longer-term follow-up studies may facilitate the delineation of a clear cutoff point for determining the efficacy of treatment in achieving a “true cure” in cases of recurrence.

Although *BRCA* mutations are associated with a favorable prognosis in ovarian cancer [25], data on the cure rate after long-term follow-up of recurrent cases are limited. A recent Japanese cohort study reported tumor *BRCA* mutations in 29% of HGSCs, 4% of clear cell carcinomas, and 19% of endometrioid carcinomas [26]. In the present study, among cases that remained disease-free for an extended period after recurrence, *BRCA* mutations were found in 45% (5/11) of HGSC cases and 0% (0/6) of cases of other histologic types, indicating no significant difference compared to the general population. DNA homologous recombination repair deficiency caused by *BRCA* mutations leads to intratumoral heterogeneity [27], which may impede complete eradication of the disease.

In this analysis, most of the patients were Japanese. Among patients with ovarian cancer, Asians have been reported to have a better prognosis [28], but also higher sensitivity to chemotherapy [29]. In addition, Asians may have an increased sensitivity to PARP inhibitors [30]. Other multicenter retrospective studies conducted in Japan [13] have reported similar recurrence rates (18.5% recurrence rate among 675 cases), which is consistent with the present study. Therefore, the results of this study may not be directly generalizable to non-Asian populations.

The limitations of this study include its retrospective nature, data collection from a limited number of institutions, and the long study period, during which the introduction of bevacizumab and PARP inhibitors resulted in a heterogeneous population. Therefore, it is important to acknowledge that the emergence of discrepancies in drug therapy among groups, as illustrated in Table 1, does not inherently suggest superiority or inferiority in terms of efficacy. In addition, many of the FFPE samples were old and had poor DNA quality, which prevented *BRCA* mutation analysis of the tumors in a significant number of cases (13/30; 43%). In the PAOLA-1 study, of 142 cases with unknown HRD status, 109 (77%) were known to have the *BRCA* wild type, but their genomic instability score remained unknown [31]. This suggests that when DNA quality is poor, genomic scars may be more difficult to detect than *BRCA* mutations. It is difficult to characterize the genome of tumors that are cured after recurrence, and it is difficult to do so retrospectively after a cured patient has been identified. Furthermore, due to limitations in the number of cases and research methodology, analytical techniques such as recursive partitioning analysis could not be performed. Large-scale prospective studies are needed to address the issues in this study. The results of such research could enable the prediction of prognosis based on molecular patterns observed at the initial diagnosis.

## 5. Conclusions

Cases of ovarian cancer that retained NED status for a long period after recurrence had a lower ascites volume at the initial diagnosis, longer DFS, higher incidence of isolated recurrence, and a higher rate of complete SDS. Regardless of the number of recurrences, ovarian cancer could be considered cured if DFS reached 4 years. Among cases of CR after recurrence, 19.4% achieved 4-year DFS, which corresponds to 9.3% of recurrent cases of ovarian cancer. There were no significant differences in the *BRCA* mutation rate in these cases compared to previous reports, and there were no *BRCA* mutations in non-HGSC cases, which highlights the need for further investigation into the factors that contribute to patients being cured after recurrence.

## Figures and Tables

**Table 1:** Recurrent ovarian cancer in a university group. Recurrent cases that achieved CR after debulking surgery and chemotherapy but subsequently recurred are shown. The three groups were compared at the time of the final survey: those who did not achieve CR after recurrence (non-CR), those who achieved CR but were dead of disease (DOD) or alive with disease (AWD), and those who had no evidence of disease (NED). For treatment of recurrent tumors, only the two groups that achieved a CR after a recurrence were compared.

	Non-CR	DOD /AWD	CR-NED	Univariate *p* Value	Multivariate *p* Value
number of cases	84	54	21		
Characteristics at diagnosis					
median Age y.o.	59.5	59.5	60.0	0.91	0.42
Diabetes Mellitus (%)	3 (4)	3 (6)	0 (0)	0.72	
Hypertension (%)	19 (23)	8 (15)	3 (14)	0.49	
median BMI	20.8	21.1	21.5	0.42	
FIGO Stage III/IV (%)	68 (81)	49 (91)	16 (76)	0.21	0.96
Metastatic site	Distant LNs.: 6 Distant Organ: 5	Distant LNs.: 2 Distant Organ: 1	Distant LNs.: 2 Distant Organ: 1		
Serous carcinoma (%)	59 (70)	41 (76)	17 (81)	0.76	0.44
median CA125 IU/L	762.5	308.0	634.7	0.14	0.87
ascites > 200 mL (%)	39 (46)	13 (28)	2 (12)	0.001	0.02
Treatment of primary tumor					
NAC-IDS (%)	40 (48)	28 (52)	10 (48)	0.99	0.55
complete surgery (%)	52 (62)	37 (70)	16 (76)	0.65	0.50
fertility preservation (%)	0 (0)	2 (4)	1 (5)	0.13	0.28
no chemotherapy (%)	0 (0)	0 (0)	3 (14)	0.002	0.99
Disease status at first recurrence					
median DFS1 mo.	8.0	13.0	22.8	<0.001	<0.001
median CA125 IU/L	96.0	51.0	75.5	0.04	0.34
solitary lesion (%)	10 (12)	15 (29)	11 (52)	0.004	0.001
ascites >200 mL (%)	6 (7.1)	5 (10)	0 (0)	0.47	0.85
Treatment of recurrent tumor					
complete surgery (%)		19 (35)	13 (62)	0.04	
PARP inhibitor (%)		11 (20)	4 (20)	1.00	
Bevacizumab		11 (20)	2 (10)	0.33	

y.o.: years old. mo.: months. BMI: Body Mass Index. LNs.: Lymph nodes.

**Table 2:** Details of mutations in the five cases with tumor-BRCA pathogenic variants.

Case	Gene	Coding	Amino Acid Change	Variant Effect
c	*BRCA2*	c.6952C>T	p.Arg2318Ter	nonsense
d	*BRCA2*	c.6405_6409del	p.Asn2135Lysfs*3	frameshift deletion
i	*BRCA2*	c.6952C>T	p.Arg2318Ter	nonsense
j	*BRCA1*	c.3719_3720insA	p.Ser1241ValfsTer3	frameshift insertion
o	*BRCA1*	c.2192_2196delAAGAA	p.Lys731ArgfsTer7	frameshift deletion

## Data Availability

The data used and analyzed during the current study are available from the corresponding authors on reasonable request.

## Supplementary Materials

The following supporting information can be downloaded at: https://www.mdpi.com/article/10.3390/cancers17183069/s1, Figure S1: Prognosis of epithelial ovarian cancer in university hospital group (A) RFS after initial treatment, (B) OS after initial debulking surgery; Figure S2: Observation period for the 21 cases of CR-NED; Figure S3: Changes in DFS in recurrent cases. (A) Comparison of DFS 1 (DFS to first recurrence) and DFS 2 (DFS to second recurrence). (B) Comparison of DFS 2 (DFS to second recurrence) and DFS 3 (DFS to third recurrence). The red line indicates cases where DFS3 was prolonged by maintenance therapy; Figure S4: Similar analyses are presented for high-risk histological types (serous carcinoma, clear cell carcinoma) among cases of recurrence; Figure S5: Selection Method for the 30 Cases Obtaining FFPE Specimens. Among 2191 epithelial ovarian cancer cases, 74 cases were NED after recurrence, and FFPE specimens were collected from the 30 cases that met the following criteria: (i) cases with DFS ≥ 4 years, (ii) no history of PARP or aromatase inhibitors, (iii) FFPE specimen available; Figure S6: CPR outcome heatmap; Table S1: Background of 645 patients who underwent debulking surgery at a university hospital group; Table S2: Subset of cases from Table 1 restricted to histologically high-risk histotypes (serous carcinoma and clear cell carcinoma).

## Abbreviations

The following abbreviations are used in this manuscript:

CR	complete remission
NED	no evidence of disease
DFS	disease-free survival
HGSC	high-grade serous carcinoma
PARP	poly (ADP-ribose) polymerase
NAC	neoadjuvant chemotherapy
IDS	interval debulking surgery
SDS	secondary debulking surgery
AWD	alive with disease
DOD	died of disease
FFPE	Formalin-fixed paraffin-embedded
CPR	central pathology review
RFS	recurrence-free survival
OS	overall survival
