# Supplementary material for: Cure of Recurrent Ovarian Cancer: A Multicenter Retrospective Study
Source: Cancers (Basel). 2025 Sep 19;17(18):3069. doi: 10.3390/cancers17183069 (PMC12468123; doi:10.3390/cancers17183069)
Supplement: Supplementary file 1 [file cancers-17-03069-s001.zip › cancers-3812413-supplementary.pdf]

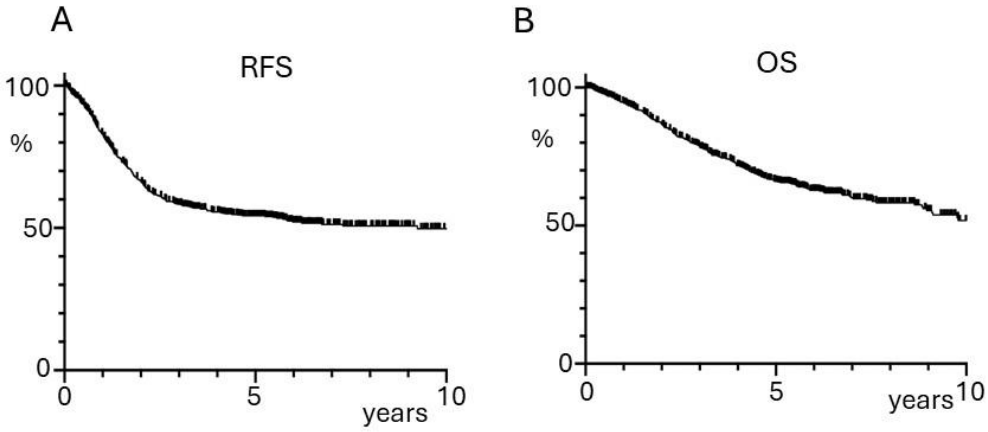

Figure S1. Prognosis of epithelial ovarian cancer in university hospital group
A) RFS after initial treatment
B) OS after initial debulking surgery

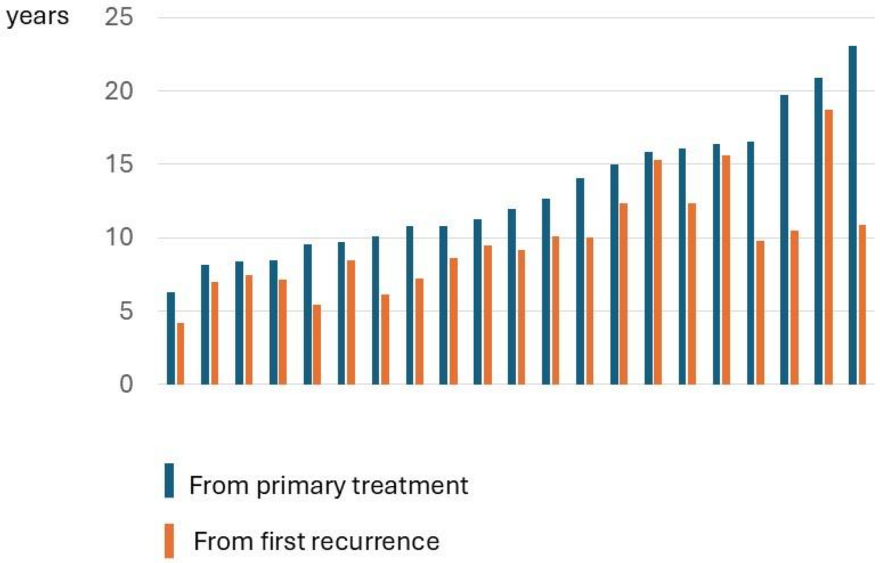

Figure S2. Observation period for the 21 cases of CR-NED

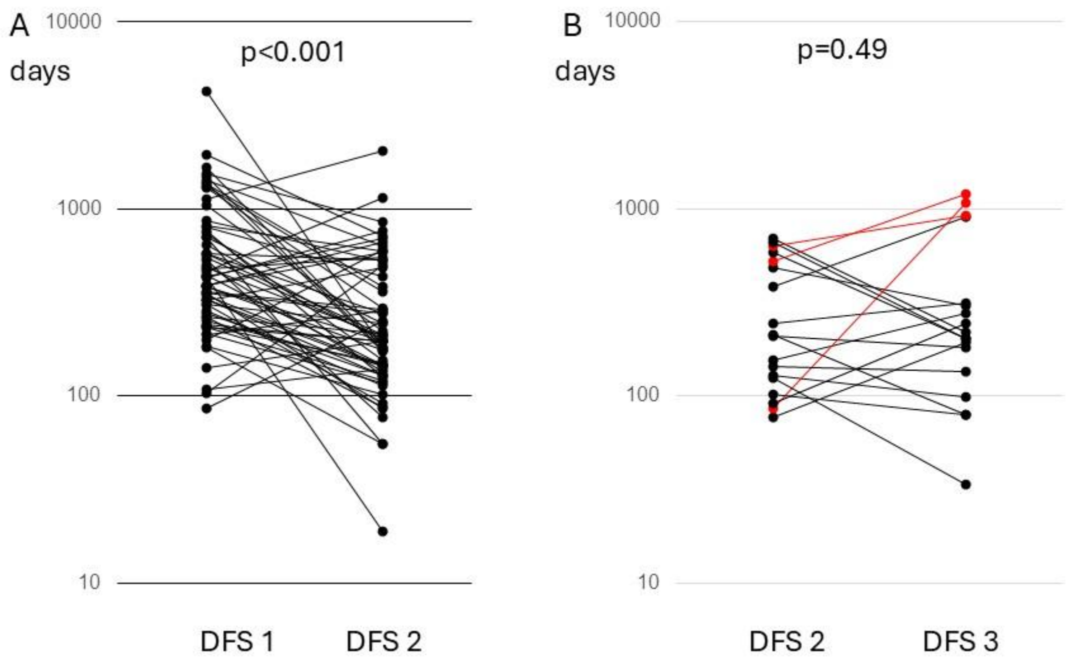

Figure S3. Changes in DFS in recurrent cases

A) Comparison of DFS 1 (DFS to first recurrence) and DFS 2 (DFS to second recurrence)

B) Comparison of DFS 2 (DFS to second recurrence) and DFS 3 (DFS to third recurrence)

The red line indicates cases where DFS3 was prolonged by maintenance therapy.

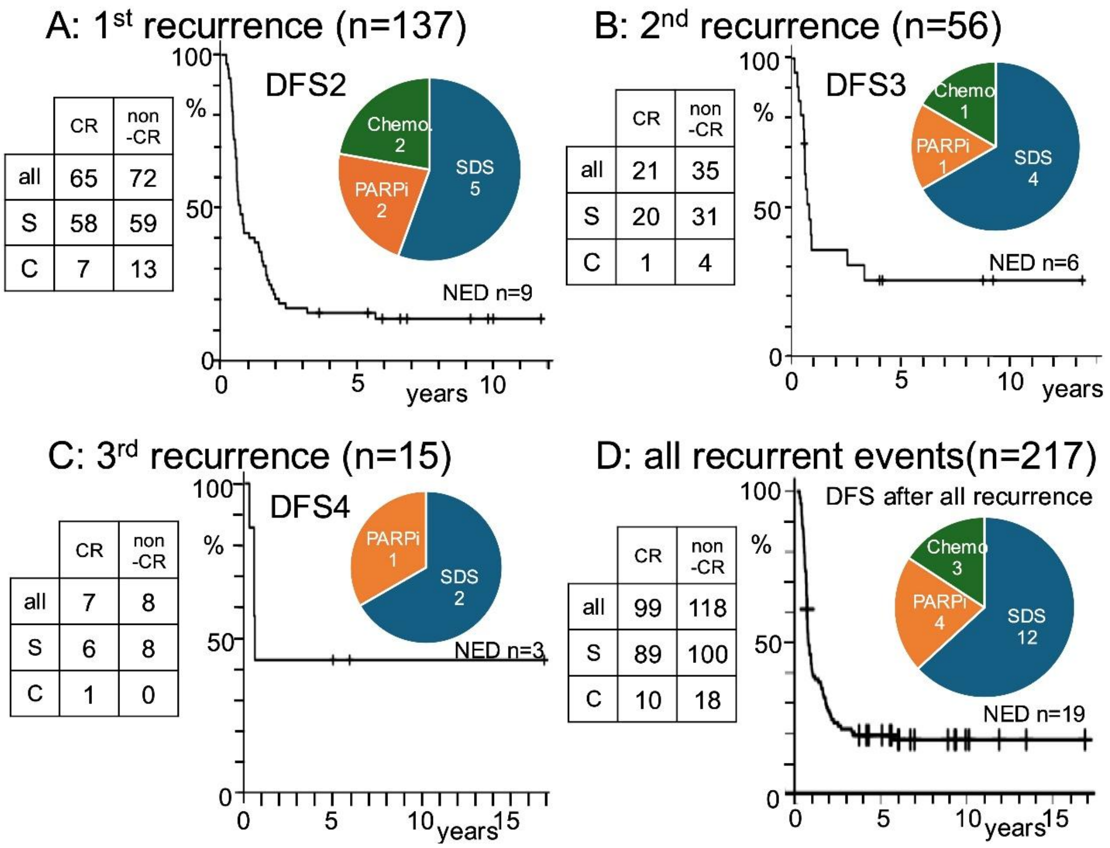

Figure S4. Similar analyses are presented for high-risk histological types (serous carcinoma, clear cell carcinoma) among cases of recurrence

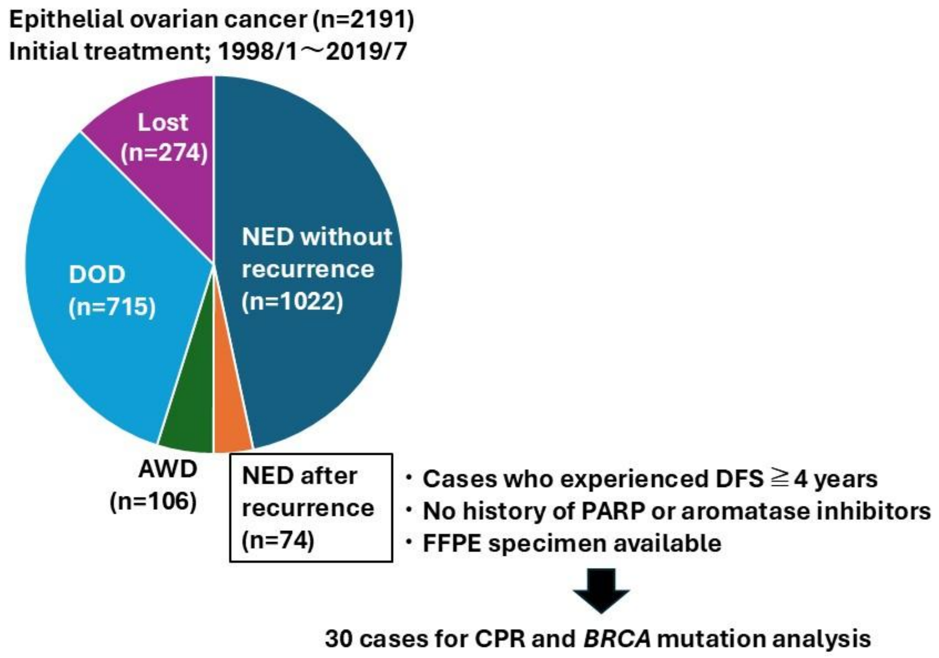

Figure S5. Selection Method for the 30 Cases Obtaining FFPE Specimens

Among 2191 epithelial ovarian cancer cases, 74 cases were NED after recurrence, and FFPE specimens were collected from 30 cases meeting the following criteria: (i) cases with DFS ≧ 4 years, (ii) no history of PARP or aromatase inhibitors, (iii) FFPE specimen available.

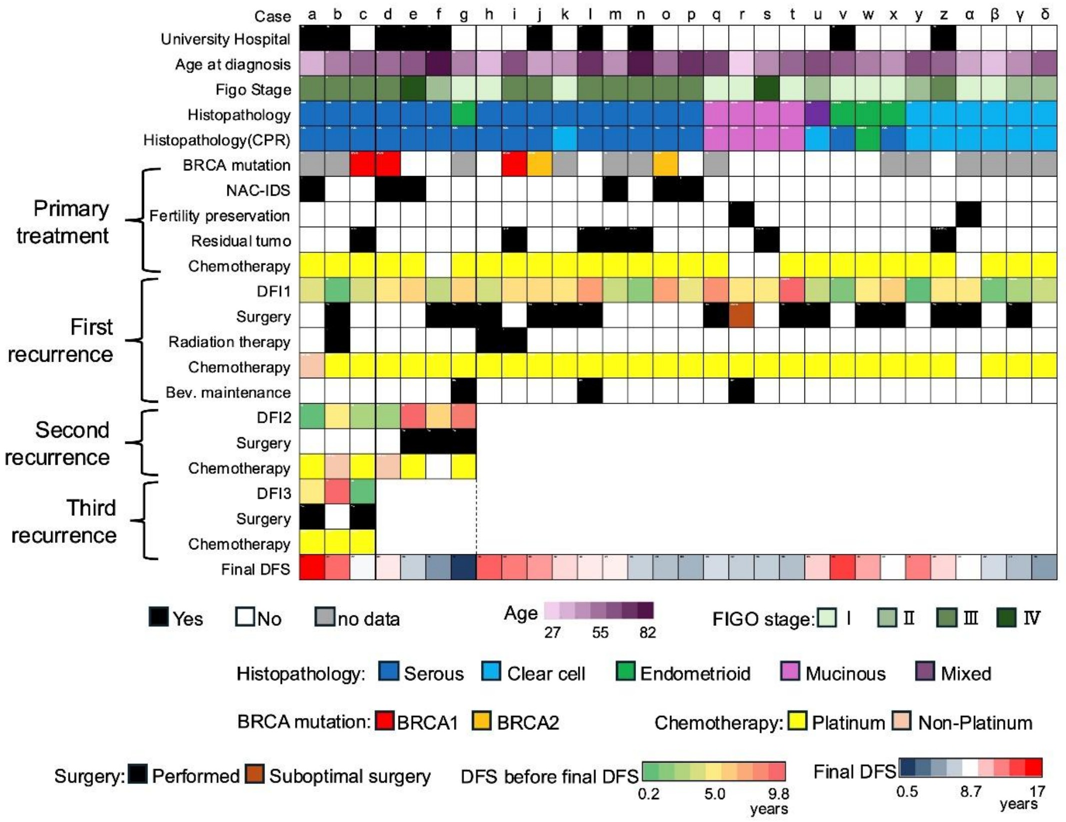

Figure S6. CPR outcome heatmap.

Age(Mean ± SD)		56.3 ± 12.0
Stage(%)		
	1	246 (40.9)
	2	43 (6.7)
	3	245(38.0)
	4	89(13.8)
Histology(%)		
	Serous	301(46.7)
	Clear	162(25.1)
	Endometrioid	86(13.3)
	Mucinous	76(11.8)
	Other	20(3.1)
PDS		451(69.9)

Table S1 Background of 645 patients who underwent debulking surgery at a university hospital group

	non-CR	DOD /AWD	CR-NED	Univariate P value	Multivariate P value
number of cases	72	47	19		
**Characteristics at diagnosis**					
median Age y.o.	58.5	60	62	0.93	0.31
Diabetes Mellitus (%)	2 (3)	3 (6)	0 (0)	0.44	
Hypertension (%)	15 (21)	7 (15)	3 (16)	0.75	
median BMI	20.7	21.4	21.5	0.63	
FIGO Stage III/IV (%)	62 (86)	44 (94)	16 (82)	0.38	0.03
Metastatic site	Distant LNs.: 6 Distant Organ: 4	Distant LNs.: 2 Distant Organ: 1	Distant LNs.: 2 Distant Organ: 1		
median CA125 IU/L	899	441	675	0.98	0.92
ascites >200ml (%)	34 (51)	11 (27)	2 (13)	<0.01	<0.01
**Treatment of primary tumor**					
NAC-IDS (%)	37 (51)	26 (55)	10 (53)	0.94	0.58
complete surgery (%)	48 (66)	32 (70)	14 (74)	0.90	0.85
fertility preservation (%)	0 (0)	0 (0)	1 (5)	0.14	0.75
no chemotherapy (%)	0 (0)	0 (0)	1 (5)	0.14	0.75
**Disease status at first recurrence**					
median DFS1mo.	7.8	14.7	23.1	<0.001	0.14
median CA125 IU/L	105	61.4	87.0	0.27	0.09

solitary lesion (%)	7 (10)	9 (20)	11 (58)	<0.001	0.19
ascites >200ml (%)	6 (9)	5 (11)	0 (0)	0.39	0.31
**Treatment of recurrent tumor**					
complete surgery (%)		14 (88)	12 (100)	0.49	
PARP inhibitor (%)		10 (21)	5 (26)	0.75	
Bevacizumab		11 (23)	2 (11)	0.32	

y.o.: years old. mo.: months. BMI: Body Mass Index. LNs.: Lymph nodes.

Table S2 Recurrent ovarian cancer histologically high-risk (serous carcinoma, clear cell carcinoma) in a university group.
